# Surgical tray leaning: carbon, efficiency and cost-savings in MAKO robotic-assisted total knee arthroplasty

**DOI:** 10.1308/rcsann.2024.0114

**Published:** 2025-03-04

**Authors:** G Al-Abbasi, C Brennan, N Ohly, C Gee

**Affiliations:** Golden Jubilee University National Hospital (GJUNH), UK

**Keywords:** Sustainability, Orthopaedics, Lean, Knee, MAKO

## Abstract

**Introduction:**

Robotic-assisted total knee arthroplasty (RA-TKA) is associated with a higher carbon footprint compared with manual TKA. This review sought to reduce the carbon and financial costs associated with MAKO RA-TKA by ‘leaning’ surgical trays.

**Methods:**

Surgeons routinely performing MAKO RA-TKA were consulted, and a consensus was reached on items from the standard knee instrument trays that were redundant and could be removed. Two new ‘lean trays’ were then introduced for MAKO RA-TKA. Carbon and financial savings were calculated based on the reduction in the number of trays requiring decontamination, sterilisation and repackaging.

**Results:**

Implementing lean methodology has reduced the tray count by one, by removing 36 out of 152 instruments per case. In five months, the use of lean trays resulted in saving 115 trays being opened, reprocessed and sterilised. This project has resulted in numerous benefits, including a total carbon saving of 220.85kgCO2e (carbon dioxide equivalent) due to reduced use of sterilisation processes (176kgCO2e) and tray wraps (44.85kgCO2). Staff feedback was positive, noting the ability to count instruments more quickly, increased space in theatre and reduced learning curve for new staff. Additionally, there was a financial saving of approximately £5,750 due to reduced burden on sterilisation services.

**Conclusions:**

It is imperative that innovative technologies are implemented with sustainability in mind and that any potential environmental harm is mitigated wherever possible. In this regard, the implementation of ‘lean’ surgical instrument trays should be considered to minimise the environmental impact of surgery while also improving efficiency and lowering costs.

## Introduction

Climate change is a crisis that causes global warming, variation in infectious diseases, pollution, resource scarcity, environmental disasters and biodiversity loss.^1^ The healthcare sector has a significant carbon footprint that contributes to greenhouse gas emissions and worsens climate change.^2^ In the UK, the NHS is responsible for approximately 5% of the country’s greenhouse gas emissions, which translates to an estimated 25 megatonnes of carbon dioxide equivalent (CO2e) per year.^3^ Operating theatres generate between 20% and 33% of a hospital’s total waste.^4^

Total knee arthroplasty (TKA) is the third most performed operation in the UK, with over 100,000 performed per annum.^5^ Each TKA produces emissions of 190.5kgCO2, equivalent to a single plane’s emission travelling from northern Scotland to the South coast of England.^1^ The MAKO robotic-assisted platform (Stryker, Mahwah, NJ, USA) has been available for TKA since 2016. Since then, the volume of TKA performed yearly has increased by 28%.^6^ The proposed advantages of robotic-assisted TKA (RA-TKA) are greater alignment accuracy, and optimising component positioning based on physiological soft tissue balancing.^7^ Whereas all these advantages may improve patients’ clinical and functional outcomes, they are associated with a relatively higher cost.^6^

The cost-effectiveness of RA-TKA is heavily determined by the volume of cases. Previous health economic studies employing the Markov model found that a certain number of annual RA-TKA would make the system cost-effective based on quality-adjusted life year (QALY) willingness to pay (WTP) calculations.^8,9^ There is also a higher environmental impact than conventional TKA (CO-TKA), although there is currently no precise record of CO2 emission and waste production.^9^ The additional equipment required for RA-TKA, which is stored in separate trays, the longer operation time, the utilisation of more drapes for the robotic arm^6^ and the need for a pre-operative computed tomography scan (emissions 9.2kgCO2e per scan) all contribute to the additional environmental impact associated with RA-TKA.^10^

Numerous strategies have been implemented globally to improve the cost-effectiveness and sustainability of the healthcare sector, with one such method being streamlining pathways and techniques. This involves a continuous process of improvement by implementing small changes focused on eliminating waste and maximising the quality of care. In the operating theatre environment, the standardisation of protocols minimises variation and enhances perioperative efficiency. Examples of adopting streamlined techniques include, but are not limited to, the use of customised surgical trays by removing frequently unused instruments to reduce theatre waste.^11,12^ Using streamlined surgical trays in orthopaedics by consolidating instrument sets has provided significant short- and long-term cost savings, improved sustainability and decreased carbon footprint.^11,13^

Despite limited literature on the sustainability of this relatively new technique, interest in this topic is growing rapidly. The Royal College of Physicians in the UK now considers it one of the seven domains of quality.^14^ Sustainability should be considered actively when implementing innovative technologies.^15^

The Golden Jubilee University National Hospital (GJUNH) is home to one of the largest elective orthopaedic centres in Europe. It is one of two NHS providers in Scotland performing MAKO RA-TKA, with two MAKO robotic arms currently in use by eight surgeons.^16^

We aimed to reduce the carbon and financial costs associated with RA-TKA by ‘leaning’ surgical trays in our department, removing items that were used infrequently or rarely to reduce the number of trays requiring processing and sterilisation.

## Methods

The contents of the standard knee instrument trays currently in use were recorded. Surgeons routinely performing RA-TKA were consulted to reach a consensus on which instruments were essential and which could be removed. The intention was to avoid additional individually sterilised and packaged supplementary items, since these create a further environment burden, neutralising any benefit from creating leaner trays. Three new ‘streamlined trays’ were then introduced for MAKO RA-TKA performed in our hospital.

We consulted with colleagues in the Central Sterile Processing Department (CSPD) to understand the pathway for the processing of our trays.

**Figure 1:**
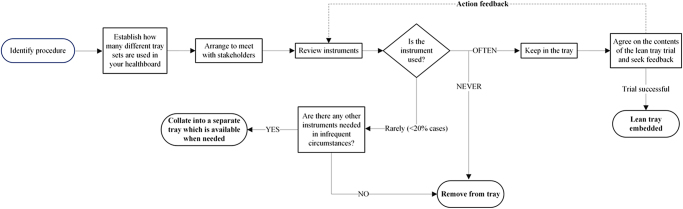
Flowchart demonstrating the process of implementing lean methodology on surgical trays

Carbon and financial savings were calculated based on the reduction in the number of trays requiring decontamination, sterilisation and repackaging. We have used data from Rizan *et al*^13^ to calculate approximate figures of CO2 emission and cost-savings associated with leaning surgical trays in RA-TKA as our sterilisation services employed similar processes. Figure 1 shows a flowchart demonstrating the process of implementing lean methodology on surgical trays.

## Results

A total of 394 RA-TKA were performed at GJUNH in 2023. The new streamlined ‘lean’ trays were introduced in September 2023. During the subsequent five-month review period, 210 RA-TKA were carried out, in which 120 cases (57.14%) were performed with the lean instrument sets. At present, three out of the ten instrument sets in use have been streamlined.

### Implementing lean methodology

Since the introduction of MAKO RA-TKA at the GJUNH in 2019, the same three knee instrument trays have been used as those required for CO-TKA, as well as two supplementary MAKO-specific trays. It was evident that many of the instruments on the conventional tray were seldom or never used during RA-TKA. This was discussed at a departmental level, and an agreement was reached to eliminate unnecessary instruments and implement lean methodology. As a result, the overall count of instrument trays has been reduced by 20% (five trays down to four), and the number of instruments reduced by 36 out of 152 (23.6%).

**Figure 2:**
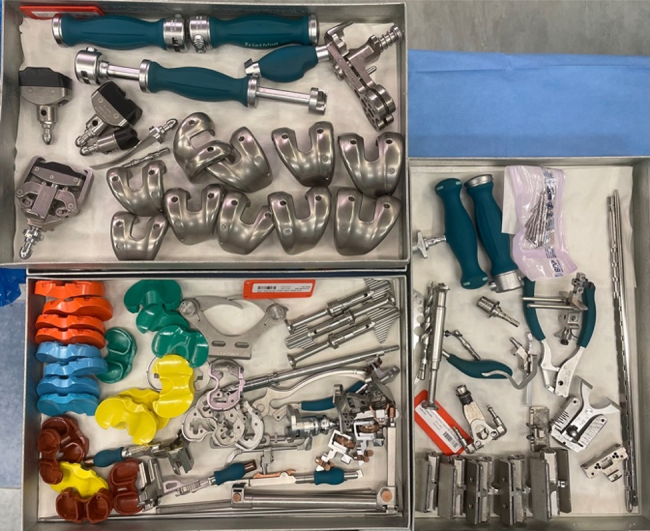
Three conventional trays before application of leaning methodology

**Figure 3:**
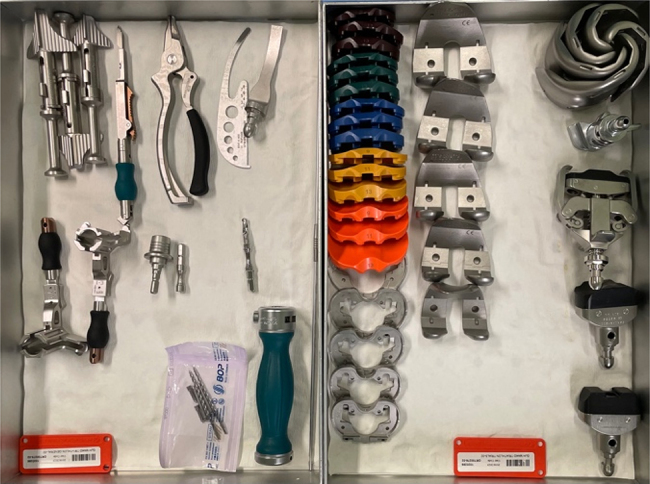
Two streamlined trays after application of leaning methodology

To ensure there are no patient safety issues, the instruments removed have been compiled into a separate ‘conversion’ tray that is available in the unlikely event of intra-operative conversion from RA-TKA to CO-TKA. Conversion trays have been requested five times (4%) since they were introduced due to perceived technical problems, which is likely to be reduced further with ongoing improvement in the learning curve. Figures 2 and 3 compare the two sets before and after the application of lean methodology.

### Improvement in the surgical trays’ sterilisation process

At our hospital, all surgical instruments are decontaminated and sterilised in the CSPD. The CSPD utilises a washer disinfector, which has the capacity for up to eight trays at a time, requiring one hour for completion. Once cleaned, the trays are passed through a steam steriliser, which can hold up to ten trays and requires an additional hour for the sterilisation process to complete.

Theoretically, with only four trays, it would be possible for the machines to accommodate two full sets simultaneously; however, in real-world scenarios, trays are processed in order of priority alongside the trays for other surgical specialities. Nevertheless, reducing the total volume of trays processed by CSPD represents a significant efficiency gain since the time saved by staff can be used to process other higher-priority trays, and fewer trays lead to fewer journeys transporting equipment to and from theatres. The environmental impact is also significant by reducing the use of resources required to process fewer instruments and trays.

### Improvement in sustainability and cost-effectiveness

The amount of CO2 emitted associated with the washing and sterilisation of one tray is approximately 1.531kgCO2e.^13^ The ‘Streamlined Sets’, which contained four trays rather than five, were used 120 times, with a fifth conversion tray opened in only 4% of cases. In the five months since their introduction, the volume of trays processed has been reduced by 115, providing a total carbon saving of 176kgCO2e (1.531kgCO2e×115) solely from avoidance of sterilisation processes.

The hospital employs a single-use interleaved green and blue ‘CLINPAK CHOICE WRAP’ consisting of four layers of polypropylene fibres with inner melt-blown layers weighing 66g and sizing 100×120cm (55gsm) for wrapping the trays. Additionally, a purple ‘CLINPAK TRANSPORT WRAP’ weighing 60g and sizing 100×120cm (50gsm) is used for transportation purposes. Using the *Greenhouse gas reporting: conversion factors 2023* document, we calculated the carbon emission per one inner wrap is 0.205kgCO2e and per transport wrap is 0.186kgCO2. By decreasing the volume of trays processed and, therefore, the use of single-use wraps, 0.39kgCO2e was saved per case, a total of 44.85kgCO2e following the implementation of streamlined trays.^16, 17^

Overall, the new streamlined trays for use in RA-TKA led to a total carbon reduction of 220.85kgCO2e and saved the hospital approximately £50 per case by avoiding the use of excess decontamination services and wraps,^18^ which was a total of approximately £5,750 during the five months post-intervention. It is worth noting that the cost savings in our study associated with RA-TKR do not necessarily result in lower overall expenses when compared with CO-TKR. Instead, these cost savings serve primarily to offset the additional costs associated with implementing robotic technology.

### Satisfaction of the surgical workforce

Stakeholders, including surgeons, surgical assistants, nurses and the Central Sterile Processing Department (CSPD), provided valuable feedback via an electronic form to assess their satisfaction and opinion on the change. Over the course of one week, 16 responses were collected, including 11 from surgeons who were routinely involved in robotic knee arthroplasty (RA-TKA). All participants were somewhat or very concerned regarding the impact the delivery of patient care has on the environment; 87.5% expressed positive feedback regarding the implementation of streamlined trays in RA-TKA, whereas one respondent remained neutral. The staff highlighted several positive consequences of the initiative, including the ability to count instruments more quickly, more working space in the theatre, reducing the physical burden of handling instruments and lowering the learning curve for new scrubbing staff.

## Discussion

Although one might infer that reducing the number of instruments is key to reducing carbon emissions, the true objective is reducing the number of trays being processed. When instruments are removed from sets, they may be supplied as individually wrapped supplementals with a two- to three-fold higher carbon footprint compared with an instrument in a tray set when they are opened,^13^ as well as greater decontamination and sterilisation services labour. If multiple supplemental instruments are opened frequently, the carbon footprint may be conversely increased compared with conventional tray sets. It has been suggested previously in the literature that instruments should be removed from tray sets only if they are used in less than 20% of cases.^19^

When obtaining additional instruments during surgery, the carbon footprint of decontamination and packaging was lower when ten or fewer additional individually wrapped instruments were opened. The costs were lower when four or fewer items were required. Above these thresholds, carbon and financial costs were lower when instruments were obtained by opening an additional set.^13^

Our centre performed 394 RA-TKA in 2023, with increasing volumes projected. If conversion trays are requested in 4% of cases, we anticipate a total carbon reduction of at least 726.1kgCO2e and financial savings in the region of at least £18,900 per annum with the expansion of lean trays for all RA-TKA cases. When introducing lean trays, it is vital to engage the relevant stakeholders, including surgeons, sterilisation and processing services, scrub staff and theatre stores. If instruments are used in less than 20% of cases, then removing them from instrument trays has demonstrable financial savings. Figure 3 presents a flowchart of the process of implementing the leaning methodology on surgical instruments.

## Conclusions

Innovative technologies must be implemented with sustainability in mind, and environmental harm must be mitigated where possible. Lean methodology in the form of streamlining surgical trays should be considered in more orthopaedic procedures and in other specialities to reduce the impact of surgical procedures on the environment and improve theatre efficiency. In our department, we plan to expand on this work further by commencing the ‘Leaning’ of other commonly used tray sets. This work has been highlighted by the National Green Theatres Programme and lessons will be shared nationally to encourage other departments to review their current instrument sets and find opportunities for leaning.

## References

[1] Watts N, Amann M, Arnell N *et al.* The 2020 report of the Lancet countdown on health and climate change: responding to converging crises. *Lancet* 2021; **397**: 129–170. 33278353 10.1016/S0140-6736(20)32290-X PMC7616803

[2] Albert MG, Rothkopf DM. Operating room waste reduction in plastic and hand surgery. *Plast Surg* 2015; **23**: 235–238. 10.4172/plastic-surgery.1000941 PMC4664137 26665137

[3] Tennison I, Roschnik S, Ashby B *et al.* Health care’s response to climate change: a carbon footprint assessment of the NHS in England. *Lancet Planet Health* 2021; **5**: e84–e92. 33581070 10.1016/S2542-5196(20)30271-0 PMC7887664

[4] Kagoma Y, Stall N, Rubinstein E *et al.* People, planet and profits: the case for greening operating rooms. *CMAJ* 2012; **184**: 1905–1911. 22664760 10.1503/cmaj.112139 PMC3503903

[5] Royal College of Surgeons of England. *Surgery and the NHS in numbers*. https://www.rcseng.ac.uk/news-and-events/media-centre/media-background-briefings-and-statistics/surgery-and-the-nhs-in-numbers/ (cited April 2024).

[6] Kolessar DJ, Hayes DS, Harding JL *et al.* Robotic-arm assisted technology’s impact on knee arthroplasty and associated healthcare costs. *J Health Econ Outcomes Res* 2022; **9**: 57. 36072348 10.36469/001c.37024 PMC9398468

[7] Batailler C, Fernandez A, Swan J *et al.* MAKO CT-based robotic arm-assisted system is a reliable procedure for total knee arthroplasty: a systematic review. *Knee Surg Sports Traumatol Arthrosc* 2021; **29**: 3585–3598. 32975626 10.1007/s00167-020-06283-z

[8] Rajan PV, Khlopas A, Klika A *et al.* The cost-effectiveness of robotic-assisted versus manual total knee arthroplasty: a Markov model–based evaluation. *JAAOS-J Am Acad Orthop Surg* 2022; **30**: 168–176. 10.5435/JAAOS-D-21-00309 35040808

[9] Yasen Z, Woffenden H, Robinson AP. Robotic-assisted knee arthroplasty: insights and implications from current literature. *Cureus* 2023; **15**: e50852. 38249205 10.7759/cureus.50852 PMC10798799

[10] McAlister S, McGain F, Breth-Petersen M *et al.* The carbon footprint of hospital diagnostic imaging in Australia. *Lancet Reg Health West Pac* 2022; **24**: 100459. 35538935 10.1016/j.lanwpc.2022.100459 PMC9079346

[11] Cichos KH, Hyde ZB, Mabry SE *et al.* Optimization of orthopedic surgical instrument trays: lean principles to reduce fixed operating room expenses. *J Arthroplasty* 2019; **34**: 2834–2840. 31473059 10.1016/j.arth.2019.07.040

[12] Cichos KH, Linsky PL, Wei B *et al.* Cost savings of standardization of thoracic surgical instruments: the process of lean. *Ann Thorac Surg* 2017; **104**: 1889–1895. 29054303 10.1016/j.athoracsur.2017.06.064

[13] Rizan C, Lillywhite R, Reed M *et al.* Minimising carbon and financial costs of steam sterilisation and packaging of reusable surgical instruments. *Br J Surg* 2022; **109**: 200–210. 34849606 10.1093/bjs/znab406 PMC10364739

[14] Mortimer F, Isherwood J, Wilkinson A *et al.* Sustainability in quality improvement: redefining value. *Future Healthc J* 2018; **5**: 88–93. 31098540 10.7861/futurehosp.5-2-88 PMC6502556

[15] Smith JT, Boakye LA, Ferrone ML *et al.* Environmental sustainability in the orthopaedic operating room. *J Am Acad Orthop Surg* 2022; **30**: 1039–1045. 36007200 10.5435/JAAOS-D-22-00247

[16] Public Health Scotland. Scottish Arthroplasty Project. https://www.publichealthscotland.scot/publications/scottish-arthroplasty-project/scottish-arthroplasty-project-12-september-2023/#:~:text=The%20independent%20sector%20is%20now,2020%20to%200.3%25%20in%202022. (cited March 2024).

[17] Gov.uk. *Greenhouse gas reporting: conversion factors 2023* https://www.gov.uk/government/publications/greenhouse-gas-reporting-conversion-factors-2023 (cited April 2024).

[18] Maidstone and Tunbridge Wells NHS Trust. *Freedom of information Act 2000.* https://www.mtw.nhs.uk/wp-content/uploads/2019/10/Decontamination-and-sterilisation-service.-300719.pdf (cited April 2024).

[19] Nast K, Swords K. Decreasing operating room costs via reduction of surgical instruments. *J Pediatr Urol* 2019; **15**: 153.e1–153.e6. 10.1016/j.jpurol.2019.01.013 30846251

